# Post-Proliferative Immature Radial Glial Cells Female-Specifically Express Aromatase in the Medaka Optic Tectum

**DOI:** 10.1371/journal.pone.0073663

**Published:** 2013-09-03

**Authors:** Akio Takeuchi, Kataaki Okubo

**Affiliations:** Department of Aquatic Bioscience, Graduate School of Agricultural and Life Sciences, the University of Tokyo, Bunkyo, Tokyo, Japan; University of Rouen, France, France

## Abstract

Aromatase, the key enzyme responsible for estrogen biosynthesis, is present in the brain of all vertebrates. Much evidence has accumulated that aromatase is highly and exclusively expressed in proliferating mature radial glial cells in the brain of teleost fish even in adulthood, unlike in other vertebrates. However, the physiological significance of this expression remains unknown. We recently found that aromatase is female-specifically expressed in the optic tectum of adult medaka fish. In the present study, we demonstrated that, contrary to the accepted view of the teleost brain, female-specific aromatase-expressing cells in the medaka optic tectum represent a transient subset of post-proliferative immature radial glial cells in the neural stem cell lineage. This finding led us to hypothesize that female-specific aromatase expression and consequent estrogen production causes some sex difference in the life cycle of tectal cells. As expected, the female tectum exhibited higher expression of genes indicative of cell proliferation and radial glial maturation and lower expression of an anti-apoptotic gene than did the male tectum, suggesting a female-biased acceleration of the cell life cycle. Complicating the interpretation of this result, however, is the additional observation that estrogen administration masculinized the expression of these genes in the optic tectum, while simultaneously stimulating aromatase expression. Taken together, these results provide evidence that a unique subpopulation of neural stem cells female-specifically express aromatase in the optic tectum and suggest that this aromatase expression and resultant estrogen synthesis have an impact on the life cycle of tectal cells, whether stimulatory or inhibitory.

## Introduction

Aromatase, the key steroidogenic enzyme responsible for the conversion of androgens to estrogens, is present in the brain of all classes of vertebrates. There is accumulating evidence that aromatase plays integral roles in the process of brain sexual differentiation in mammals and birds. This is best exemplified in perinatal male rodents, where extremely high levels of aromatase activity are transiently induced in the brain, and testosterone secreted from the testis is converted to estradiol-17β (E2) by the action of brain aromatase to organize male-type neural circuitry [1–3]. In addition, studies in aromatase-knockout mice have shown that a central expression of aromatase is essential for the development of female-typical behavior [4].

The brain of adult teleost fish exhibits 100–1000-fold greater aromatase activity than the adult mammalian and avian brain [5]. It has been suggested that this exceptionally high level of brain aromatase activity during adulthood represents a similar situation to that occurring in perinatal rodents and thus contributes to the establishment of sex differences in the teleost brain. However, there has been virtually no evidence to support this idea. Interestingly, in the teleost brain, aromatase is expressed exclusively in radial glial cells, whereas neuronal cells are its primary source in the brain of other vertebrates [6–9]. Radial glial cells have been shown to undergo proliferative and differentiating cell divisions to generate both neurons and other glial lineages in many vertebrate species including teleosts [10–12]. Studies in various teleost species have shown that aromatase-expressing cells in the brain also express glial fibrillary acidic protein (Gfap), which is a canonical marker of mature radial glial cells in teleosts, and proliferating cell nuclear antigen (Pcna), which is an established proliferation marker [6,7,12–14]. It is thus generally accepted that aromatase-expressing cells in the teleost brain are proliferating mature radial glial cells.

We and another research group have recently found that, in medaka (*Oryzias latipes*), a teleost species widely used as a model organism in reproductive biology and neuroscience studies, a gene encoding aromatase, *cyp19a1b*, is expressed more abundantly expressed in female than in male brain [15,16]. We also found that medaka *cyp19a1b* is expressed throughout the ventricular zones in wide areas of the brain, where, in most regions, females have a greater degree of expression compared to males [16]. The most prominent sex difference was observed in the ventricular grey zone (VGZ) of the optic tectum (the midbrain region homologous to the superior colliculus of mammals), where expression was confined almost exclusively to females [16]. This represents the first evidence of brain aromatase expression specific to only one sex. Intriguingly, recent evidence has indicated that proliferating cells in the teleost optic tectum constitute a cell population distinct from radial glial cells. These proliferating cells have properties similar to neuroepithelial cells [17,18], which appear transiently in mammalian embryos as precursors of radial glial cells [19]. This distinct existence of radial glial cells and proliferating cells raises concern regarding the cell population that harbors female-specific *cyp19a1b* expression in the medaka optic tectum.

In the present study, we determined the characteristics of female-specific *cyp19a1b*-expressing cells in the medaka optic tectum. Contrary to the general belief as well as our own expectations regarding aromatase-expressing cells in the teleost brain, most of these cells were found to be neither mature radial glial cells nor proliferating neuroepithelial cells, but they represented an early transient stage of radial glial cells. This finding led us to assume that aromatase expression and the resulting estrogen production are involved in the control of the cell life cycle in a sex-dependent manner in the medaka optic tectum. The results of subsequent analyses indicated that sex differences existed in the life cycle of tectal cells, and that aromatase expression and consequent estrogen synthesis, with enhancive or suppressive effects, were involved in this event.

## Materials and Methods

### Ethics statement and animals

The care and use of animals in this study were in accordance with the guidelines of the Committee on Life Sciences of the University of Tokyo. The committee requests the submission of an animal use protocol only for use of mammalian, avian, reptilian species, in accordance with the Fundamental Guidelines for Proper Conduct of Animal Experiment and Related Activities in Academic Research Institutions under the jurisdiction of the Ministry of Education, Culture, Sports, Science and Technology of Japan (Ministry of Education, Culture, Sports, Science and Technology, Notice No. 71; June 1, 2006). Therefore, we did not submit an animal use protocol for this study, which used only teleost fish species and was unnecessary to be approved by the committee.

The wild-type d-rR strain and *cyp19a1b*-GFP transgenic line (generously gifted by Dr. Ramji K. Bhandari), in which *cyp19a1b*-expressing cells are labeled with green fluorescent protein (GFP), of medaka were bred and maintained at 28 ^°^C with a 14-h light/10-h dark photoperiod. They were fed 3 or 4 times per day with live brine shrimp and commercial pellet food. Sexually mature fish of 3–4 months post-fertilization were sampled at 1–2.5 h following the onset of light and used for analyses. For sampling, fish were anesthetized by immersion in ice water and rapidly sacrificed by decapitation.

### In silico cloning of cell marker genes and cell life cycle-related genes in medaka

The expressed sequence tag (EST) database of National BioResource Project (NBRP) Medaka (http://www.shigen.nig.ac.jp/medaka/) was searched for medaka ESTs for *pcna*, *mki67* (encoding an antigen identified by monoclonal antibody Ki-67), *sox2* (encoding sex determining region Y-box 2), *msi1* (encoding RNA-binding protein Musashi homolog 1), *gfap*, *bcl2* (encoding B cell leukemia/lymphoma 2), *bcl2l1* (encoding Bcl2-like 1), *baxa* (encoding Bcl2-associated X protein, a), and *baxb* (encoding Bcl2-associated X protein, b) by using the TBLASTN program with amino acid sequences of their respective human orthologs as query sequences. This search resulted in the identification of EST clones for which full-length sequences were available for *pcna*, *sox2*, *msi1*, *gfap*, *bcl2*, *bcl2l1*, *baxa*, and *baxb*. However, only EST clones partially sequenced could be identified for *mki67*. Accordingly, the Ensembl medaka genome database (http://www.ensembl.org/Oryzias_latipes/blastview) was also searched with the BLAT program and these partial EST clones as queries to obtain longer sequence information for medaka *mki67*.

The deduced amino acid sequences from the medaka ESTs and predicted transcript identified were aligned with those of their respective counterparts in other species by using Clustal W software. A phylogenetic tree was then constructed using the neighbor-joining method (http://clustalw.ddbj.nig.ac.jp/index.php). For medaka *mki67*, the partial sequence empirically determined by us during the preparation of a probe for *in situ* hybridization as described below was used in the phylogenetic analysis. The species names and accession numbers of the sequences used in the analysis are listed in Table 1.

**Table 1:** Species names and accession numbers of the protein sequences used for phylogenetic analysis.

Protein	Species	Accession number
PCNA	Human (*Homo sapiens*)	NP_002583
PCNA	Mouse (*Mus musculus*)	NP_035175
Pcna	Chicken (*Gallus gallus*)	NP_989501
Pcna	*Xenopus* (*Xenopus laevis*)	NP_001081011
Pcna	Zebrafish (*Danio rerio*)	AAH49535
Pcna	Cichlid ( *Haplochromis* *burtoni* )	AAT78432
Pcna	Eel ( *Anguilla* *japonica* )	BAA77390
MKI67	Human (*Homo sapiens*)	NP_001139438
MKI67	Mouse (*Mus musculus*)	NP_001074586
Mki67 (predicted)	Chicken (*Gallus gallus*)	XP_421822
Mki67	*Xenopus* (*Xenopus laevis*)	AAI21193
Mki67	Seabass ( *Dicentrarchus* *labrax* )	CBN81259
SOX2	Human (*Homo sapiens*)	NP_003097
SOX2	Mouse (*Mus musculus*)	NP_035573
Sox2	Chicken (*Gallus gallus*)	NP_990519
Sox2	*Xenopus* (*Xenopus laevis*)	NP_001081691
Sox2	Zebrafish (*Danio rerio*)	NP_998283
Sox2	Salmon (*Salmo salar*)	NP_001135190
Sox2	Fugu ( *Takifugu* *rubripes* )	AAQ18495
Sox2	Tilapia ( *Oreochromis* *niloticus* )	ABO26869
MSI1	Human (*Homo sapiens*)	NP_002433
MSI1	Mouse (*Mus musculus*)	NP_032655
Msi1 (predicted)	Chicken (*Gallus gallus*)	XP_415271
Msi1	*Xenopus* (*Xenopus laevis*)	NP_001084040
Msi1	Zebrafish (*Danio rerio*)	NP_001013534
Msi1	Seabass ( *Dicentrarchus* *labrax* )	CBN81137
GFAP	Human (*Homo sapiens*)	AAB22581
GFAP	Mouse (*Mus musculus*)	AAI39359
Gfap (predicted)	Chicken (*Gallus gallus*)	XP_418091
Gfap (predicted)	Gekko ( *Gekko* *japonicus* )	ACX71855
Gfap	Zebrafish (*Danio rerio*)	NP_571448
BCL2	Human (*Homo sapiens*)	NP_000624
BCL2	Mouse (*Mus musculus*)	AAH95964
Bcl2	Chicken (*Gallus gallus*)	NP_990670
Bcl2	*Xenopus* (*Xenopus laevis*)	NP_001139565
Bcl2	Zebrafish (*Danio rerio*)	NP_001025424
Bcl2	Pejerrey ( *Odontesthes* *bonariensis* )	ACP19736
BCL2L1	Human (*Homo sapiens*)	NP_612815
BCL2L1	Mouse (*Mus musculus*)	NP_033873
Bcl2l1	Chicken (*Gallus gallus*)	NP_001020475
Bcl2l1	*Xenopus* (*Xenopus laevis*)	NP_001082147
Bcl2l1	Zebrafish (*Danio rerio*)	NP_571882
BAX	Human (*Homo sapiens*)	NP_620116
BAX	Mouse (*Mus musculus*)	NP_031553
Bax	*Xenopus* (*Xenopus laevis*)	NP_001079104
Baxa	Zebrafish (*Danio rerio*)	NP_571637
Baxb	Zebrafish (*Danio rerio*)	NP_001013314

### Sample preparation for histological analysis

Three female and 3 male fish were used for each histological analysis. Whole brains were dissected, fixed in 4% paraformaldehyde (PFA) for 3 h, and immersed sequentially in 10%, 20%, and 30% sucrose in phosphate-buffered saline (PBS). The specimens were embedded in 5% agarose (Type IX-A; Sigma-Aldrich, St. Louis, MO) in PBS supplemented with 20% sucrose, and then, they were snap-frozen in *n*-hexane at -80 ^°^C and sectioned into 20-µm (for *in situ* hybridization and immunohistochemistry) or 30-µm (for observation of GFP fluorescence) coronal slices by using a cryostat (HM 550; Thermo, Fisher Scientific, Waltham, MA). The sections of the GFP transgenic fish were coverslipped with a mounting medium containing 4′,6-diamidino-2-phenylindole (DAPI) and immediately used for microscopy.

### Double in situ hybridization

Complementary DNA fragments of 1250, 1320, and 1157 bp in length for *mki67*, *sox2*, and *msi1*, respectively, were PCR-amplified from the reverse-transcribed RNA of the medaka brain by using primers designed based on the EST sequences (Table 2). These fragments were subcloned into the pGEM-Teasy vector (Promega, Madison, WI) and sequenced in both directions with the BigDye Terminator v 3.1 Cycle Sequencing Kit on an ABI Prism 310 Genetic Analyzer (Life Technologies, Carlsbad, CA). The fragments were subsequently labeled with fluorescein (FITC) using the FITC RNA Labeling Mix and T7 RNA polymerase (Roche Diagnostics, Basel, Switzerland) to generate FITC-labeled *mki67*, *sox2*, and *msi1* antisense probes. The digoxigenin (DIG)-labeled *cyp19a1b* antisense probe was generated as previously described [16].

**Table 2:** Primers used in this study.

Target	Direction	Purpose	Sequence
*pcna*	Forward	Real-time PCR	5'-GAGGACATCATCACCCTCAGA-3'
*pcna*	Reverse	Real-time PCR	5'-TCATAATCTGAAACTTTTTCTTGGTTG-3'
*mki67*	Forward	*In situ* hybridization	5'-CGTTGAGAACTCGACGTTTTTGTG-3'
*mki67*	Reverse	*In situ* hybridization	5'-GGAGAGAGCTGGTCTTTCCTGA-3'
*mki67*	Forward	Real-time PCR	5'-ACCAATCTGAGCACAGCCAAC-3'
*mki67*	Reverse	Real-time PCR	5'-GGTGCAGGTGGATACTCAAAC-3'
*sox2*	Forward	*In situ* hybridization	5'-TTCCCTGATGTATAACATGATGGA-3'
*sox2*	Reverse	*In situ* hybridization	5'-GGAAAATAAAAATGCAACCTCTTGC-3'
*sox2*	Forward	Real-time PCR	5'-GACCCTCATGAAGAAGGACAAAT-3'
*sox2*	Reverse	Real-time PCR	5'-GGTCCCCATACCGTTTCCT-3'
*msi1*	Forward	*In situ* hybridization	5'-TCGAGGACACGGAATGGAGAC-3'
*msi1*	Reverse	*In situ* hybridization	5'-GGTGAAAGTTCAGTCATCACTGA-3'
*msi1*	Forward	Real-time PCR	5'-CAGCTGTTAGCAGTTACCTCAGC-3'
*msi1*	Reverse	Real-time PCR	5'-AAGGCTGTTGCAATCAAAGG-3'
*gfap*	Forward	Real-time PCR	5'-TGCAGGATGAGATTGTGCAG-3'
*gfap*	Reverse	Real-time PCR	5'-CTGGACACGACTGAGAGATGC-3'
*bcl2*	Forward	Real-time PCR	5'-GTCCAACGAGGAGATGAGCA-3'
*bcl2*	Reverse	Real-time PCR	5'-ATACAGCTCCACAAAGGCATC-3'
*bcl2l1*	Forward	Real-time PCR	5'-ACCGTCTACCTGGACAACCAC-3'
*bcl2l1*	Reverse	Real-time PCR	5'-GCTCTCCTGAGACCTTCTGCT-3'
*baxa*	Forward	Real-time PCR	5'-TACTGGATGTGGGCACCAA-3'
*baxa*	Reverse	Real-time PCR	5'-AGCTGCTGTCTGACCACTTCA-3'
*baxb*	Forward	Real-time PCR	5'-GCGGGGAAGATGTTTTTGAT-3'
*baxb*	Reverse	Real-time PCR	5'-TCAGGGTCCTGTAAGTTTATCCTTT-3'
*cyp19a1b*	Forward	Real-time PCR	5'-AAGAAGATGATCCAGCAAGAG-3'
*cyp19a1b*	Reverse	Real-time PCR	5'-AGCATCAGAAGAAGTAAGAAAAGTG-3'
*actb*	Forward	Real-time PCR	5'-CCCCACCCAAAGTTTAG-3'
*actb*	Reverse	Real-time PCR	5'-CAACGATGGAGGGAAAGACA-3'

Brain sections were digested with proteinase K (1 µg/ml) for 15 min at 37 ^°^C and post-fixed with 4% PFA for 10 min. Endogenous peroxidase was blocked by immersing the sections in 0.6% hydrogen peroxide in PBS for 30 min, followed by acetylation in 0.25% acetic anhydride, 0.1 M triethanolamine for 15 min. The tissues sections were then incubated overnight at 55 ^°^C with the FITC-labeled *sox2*, *msi1*, or *ki67* probe and the DIG-labeled *cyp19a1b* probe in hybridization buffer (50% formamide, 5× saline-sodium citrate (SSC), 5× Denhardt’s solution, 2 mg/ml yeast RNA, and 30 µg/ml calf thymus DNA). After washing in 5× SSC, 50% formamide for 20 min, and in 2× SSC for 2 × 20 min at 55 ^°^C, the sections were reacted overnight at 4 ^°^C with an anti-FITC antibody conjugated to horseradish peroxidase (HRP) (diluted at 1:100; PerkinElmer, Waltham, MA) and an anti-DIG antibody conjugated to alkaline phosphatase (AP) (diluted at 1:1000; Roche Diagnostics) in Tris-buffered saline (TBS) containing 1.5% blocking reagent (Roche Diagnostics) and 5 µg/ml DAPI. The anti-FITC and DIG antibodies were visualized by the TSA Plus Fluorescein System (PerkinElmer) and Fast Red (Roche Diagnostics), respectively, according to the manufacturers’ instructions.

Fluorescent images were acquired using a confocal laser scanning microscope (C1; Nikon, Tokyo, Japan). The excitation and emission wavelengths for detection were as follows: DAPI, 405 nm and 450/35 nm; FITC, 488 nm and 515/30 nm; Fast Red, 543 nm and 605/75 nm.

### Double labeling with in situ hybridization and immunohistochemistry

Acetylation, hybridization with the DIG-labeled *cyp19a1b* antisense probe, and washing were performed as described above. After blocking with PBS containing 2% normal goat serum (Vector Laboratories, Burlingame, CA) for 30 min, the tissue sections were incubated with a primary antibody in PBS containing 2% normal goat serum, 0.1% bovine serum albumin, and 0.02% keyhole limpet hemocyanin overnight at 4 ^°^C. The primary antibodies used were mouse anti-ZO1 (diluted at 1:1000; Life Technologies), rabbit anti-GFAP (diluted at 1:500; Sigma-Aldrich), mouse anti-PCNA (diluted at 1:500; Merck, Darmstadt, Germany), and rabbit anti-BLBP (diluted at 1:1000; Merck Millipore, Billerica, MA), all of which have been shown to recognize their target protein with high specificity in teleosts [6,17,18,20]. The tissue sections were then reacted overnight at 4 ^°^C with Alexa Fluor 488-conjugated goat anti-rabbit or anti-mouse secondary antibody (diluted at 1:1000; Life Technologies) and AP-conjugated DIG antibody (Roche Diagnostics) in TBS containing 1.5% blocking reagent (Roche Diagnostics) and 5 µg/ml DAPI. AP was visualized with Fast Red (Roche Diagnostics) following the manufacturer’s protocol.

Fluorescent images were obtained as described above. The excitation and emission wavelengths for Alexa Fluor 488 were 488 nm and 515/30 nm, respectively.

### Real-time PCR analysis

Whole brains were dissected from male and female medaka (n = 6 pools of 3 individuals for each sex). The optic tectum was removed from the brain as described previously [21]. Total RNA was isolated from the optic tectum using the RNeasy Plus Universal Mini Kit (Qiagen, Hilgen, Germany) and reverse-transcribed into cDNA using the SuperScript VILO cDNA Synthesis Kit (Life Technologies).

Primers for real-time PCR for *cyp19a1b* and cell life cycle-related genes, including *pcna*, *mki67*, *sox2*, *msi1*, *gfap*, *bcl2*, *bcl2l1*, *baxa*, and *baxb*, were designed using the ProbeFinder software (Roche Diagnostics). Their sequences are shown in Table 2. The real-time PCR assay was conducted in a LightCycler 480 System II (Roche Diagnostics) in a 20-µl reaction volume containing 1× LightCycler 480SYBR Green I Master (Roche Diagnostics), 0.3 µM of each primer, and 12.5 ng of transcribed RNA. The cycle conditions were as follows: 95 ^°^C for 5 min followed by 45 cycles of 95 ^°^C for 10 s, 60 ^°^C for 10 s, and 72 ^°^C for 10 s. A melting curve analysis was performed for every reaction to ensure that a single amplicon was produced in each sample. The level of β-actin (*actb*) expression in each sample was used to normalize the expression of target mRNAs. The normalized level of each target mRNA in the whole brain of male fish was arbitrarily set to 1, and the relative difference was calculated.

### Evaluation of optic tectum size

Whole brains dissected from male and female medaka (n = 5 for each sex) were fixed with Bouin’s fluid, embedded in paraffin, sectioned at a thickness of 8 µm, and Nissl-stained. The size of the optic tectum was measured by counting the number of sections containing the optic tectum and dividing it by the number of sections containing the telencephalon. The normalized size of the male tectum was arbitrarily set to 1 and used as a reference for measuring the size of the female tectum.

### Ovariectomy and estrogen treatments

The ovary was surgically removed from female fish following a previously described procedure [22]. Ovariectomized fish were immersed in 0.8% salt water containing 100 ng/ml of E2 or its vehicle alone (0.01% ethanol) for 5 days (n = 6 pools of 3 individuals for each group). The E2 concentration used was determined according to previous reports of serum E2 levels in medaka [23–25]. Sham-operated female fish (n = 6 pools of 3 individuals) were treated with vehicle in the same way. Immediately after treatment, the optic tectum was dissected and used for real-time PCR analyses to assess the effects of estrogens on the tectal expression of *cyp19a1b* and cell life cycle-related genes that showed sexually dimorphic expression (*pcna*, *mki67*, *gfap*, and *bcl2*) as described above.

### Statistical analysis

Data are presented as mean values and the standard error of the mean (SEM). Statistical analyses were performed using GraphPad Prism software (GraphPad Software, San Diego, CA). Differences between male and female fish were evaluated for statistical significance by the unpaired two-tailed Student’s *t*-test. When an F-test indicated significant differences in the variances between the sexes, Welch’s correction to the Student’s *t*-test was employed. Comparisons among the sham-operated, ovariectomized, and ovariectomized plus E2-treated groups were evaluated by one-way analysis of variance followed by Tukey’s post hoc test.

## Results

### Identification of cell marker genes and cell life cycle-related genes in medaka

Medaka cDNAs for *pcna*, *mki67*, *sox2*, *msi1*, *gfap*, *bcl2*, *bcl2l1*, *baxa*, and *baxb* were cloned *in silico* by searching medaka ESTs and genome databases. The following medaka EST clones, for which full-length sequences were available, were identified to have the highest degree of similarity to the human query sequences: olecno50_c09 for *pcna*; olbrno58_b19 for *sox2*; oleano24_p04 for *msi1*; olbrno32_j21 for *gfap*; oleano49_p02 and olecno64_j06, which shared an identical open reading frame, for *bcl2*; olteno18_m07 and olebno24_c02, which shared an identical open reading frame, for *bcl2l1*; olebno63_e12 for *baxa*; and olebno62_h21 for *baxb*. Two partially sequenced medaka EST clones, olte27h20 and olea5c15, showed the highest similarity to human MKI67. A predicted transcript harboring the sequences of these ESTs, ENSORLT00000005461, was identified in the medaka genome database. Its exact sequence was determined by PCR amplification and sequencing of a 1250-bp fragment, which was deposited in GenBank with the accession number AB775130.

**Figure 1:**
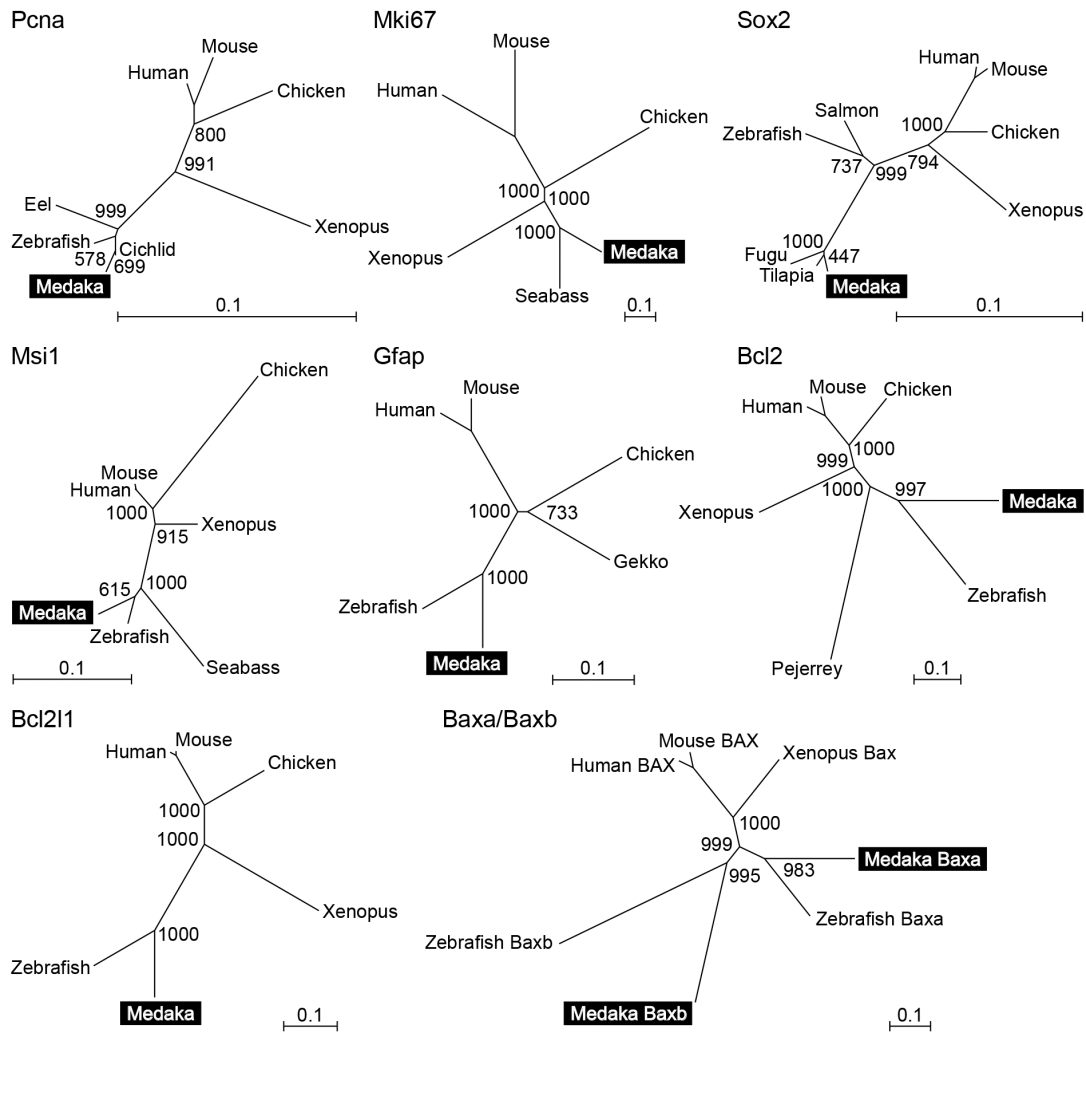
Phylogenetic analyses of genes related to the cell life cycle in vertebrates. Phylogenetic analyses of Pcna, Mki67, Sox2, Msi1, Gfap, Bcl2, Bcl2l1, and Baxa/Baxb to verify that the EST clones and predicted transcript identified in the present study encode the medaka orthologs of these proteins in other vertebrates. The number at each node indicates bootstrap values for 1000 replicates. Scale bars represent 0.1 substitutions per site.

Phylogenetic analysis provided confirmation that the EST clones and predicted transcript identified represented the medaka orthologs of *pcna*, *mki67*, *sox2*, *msi1*, *gfap*, *bcl2*, *bcl2l1*, *baxa*, and *baxb* in other vertebrates. As expected, each medaka sequence did indeed fall into the same clade as its possible orthologs in other teleost species (Figure 1).

### Majority of female-specific aromatase-expressing cells in the medaka optic tectum were neither mature radial glial cells nor proliferating cells

We next characterized the status of female-specific *cyp19a1b*-expressing cells in the medaka optic tectum by examining the expression of various cell marker genes and cell life cycle-related genes.

**Figure 2:**
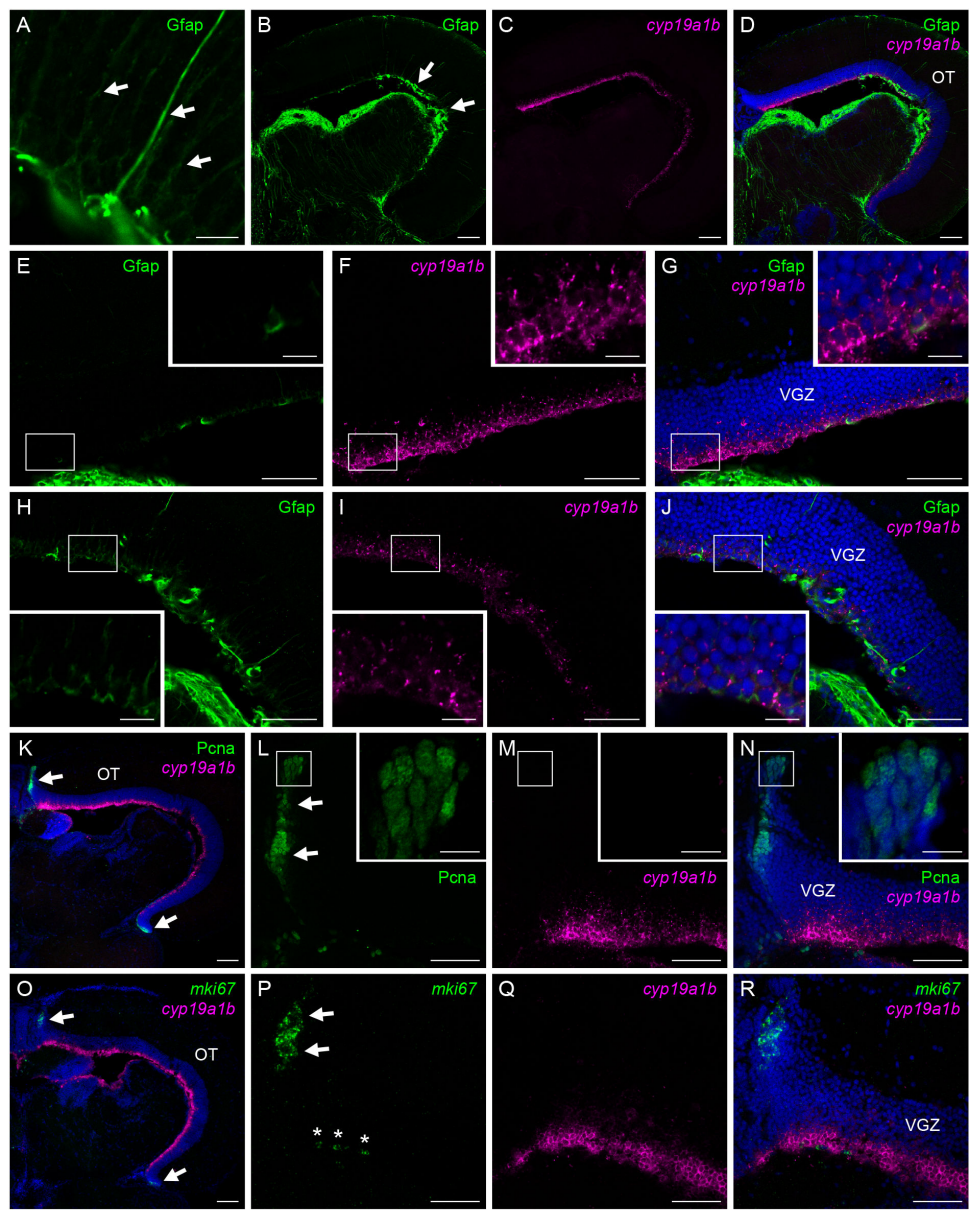
Majority of the tectal aromatase-expressing cells are neither mature radial glial cells nor proliferating cells. Absence of expression of the mature radial glial marker, Gfap (A–J) and proliferation markers, Pcna (K–N) and *mki67* (O–R), in the majority of female-specific *cyp19a1b*-expressing cells in the medaka optic tectum. (A) Gfap-positive cells exhibiting a radial glial morphology with long cytoplasmic processes (indicated by arrows). (B) Gfap expression in the whole optic tectum. Arrows indicate the central portion of the optic tectum, where Gfap expression was observed. (C) *cyp19a1b* expression in the same field as panel A. (D) Merged image of panels B and C along with DAPI staining. (E) Gfap expression in the peripheral portion of the optic tectum. (F) *cyp19a1b* expression in the same field as panel E. (G) Merged image of panels E and F along with DAPI staining. (H) Gfap expression in the central portion of the optic tectum. (I) *cyp19a1b* expression in the same field as panel H. (J) Merged image of panels H and I along with DAPI staining. (K) Expression of Pcna and *cyp19a1b* in the whole optic tectum. Arrows indicate the peripheral growth zone, where Pcna expression was detected. Nuclei were stained with DAPI. (L) Pcna expression in the peripheral growth zone (arrows). (M) *cyp19a1b* expression in the same field as panel L. (N) Merged image of panels L and M along with DAPI staining. (O) Expression of *mki67* and *cyp19a1b* in the whole optic tectum. Arrows indicate the peripheral growth zone. Nuclei were stained with DAPI. (P) *mki67* expression in the peripheral growth zone (arrows). Note that the signals indicated by asterisks lie on the dorsal surface of the cerebellum, but not in the optic tectum. (Q) *cyp19a1b* expression in the same field as panel P. (R) Merged image of panels P and Q along with DAPI staining. Small boxes in panels E–J and L–N indicate regions shown at higher magnification in the insets in the respective panels. Scale bars, 100 µm (B–D, K, O), 50 µm (E–J, L–N, P–R), and 10 µm (A, all insets). OT, optic tectum; VGZ, ventricular gray zone of the optic tectum.

Immunostaining for Gfap, an intermediate filament protein exclusively present in the radial glial cells in the teleost brain, actually labeled cells having a radial glial morphology with long cytoplasmic processes ascending toward the pial surface in the VGZ of the medaka optic tectum (Figure 2A). Gfap-positive cells were located in the ventricular surface of the VGZ, as were the *cyp19a1b*-expressing cells (Figure 2B–D). However, the Gfap-positive cells were confined to the central portion of the optic tectum (Figure 2B, arrows), whereas the *cyp19a1b*-expressing cells were broadly distributed throughout the VGZ (Figure 2C). Thus, although a small proportion of the *cyp19a1b*-expressing cells were positively labeled for Gfap, most of them were Gfap-negative. Moreover, signals for *cyp19a1b* were intense in the peripheral portion of the optic tectum, where Gfap signals were absent or sparse (Figure 2E–G), but were only modest in the central portion, where it was coexpressed with Gfap in the same cells (Figure 2H–J).

Proliferating cells identified by Pcna immunostaining were densely packed in “the peripheral growth zone”, which lies along the margin of the optic tectum, and thus did not overlap with the *cyp19a1b*-expressing cells in the ventricular surface (Figure 2K–N). The Pcna-positive cells also differed from the *cyp19a1b*-expressing cells in that they were spindle-shaped cells with elongated nuclei (Figure 2L, inset). To confirm these results, expression of another proliferation marker, *mki67*, was also analyzed. Similar to Pcna-positive cells, *mki67*-expressing cells were located in the peripheral growth zone and did not overlap with the *cyp19a1b*-expressing cells (Figure 2O–R).

### Female-specific aromatase-expressing cells in the medaka optic tectum expressed genes indicative of multipotency and exhibit cell polarity

**Figure 3:**
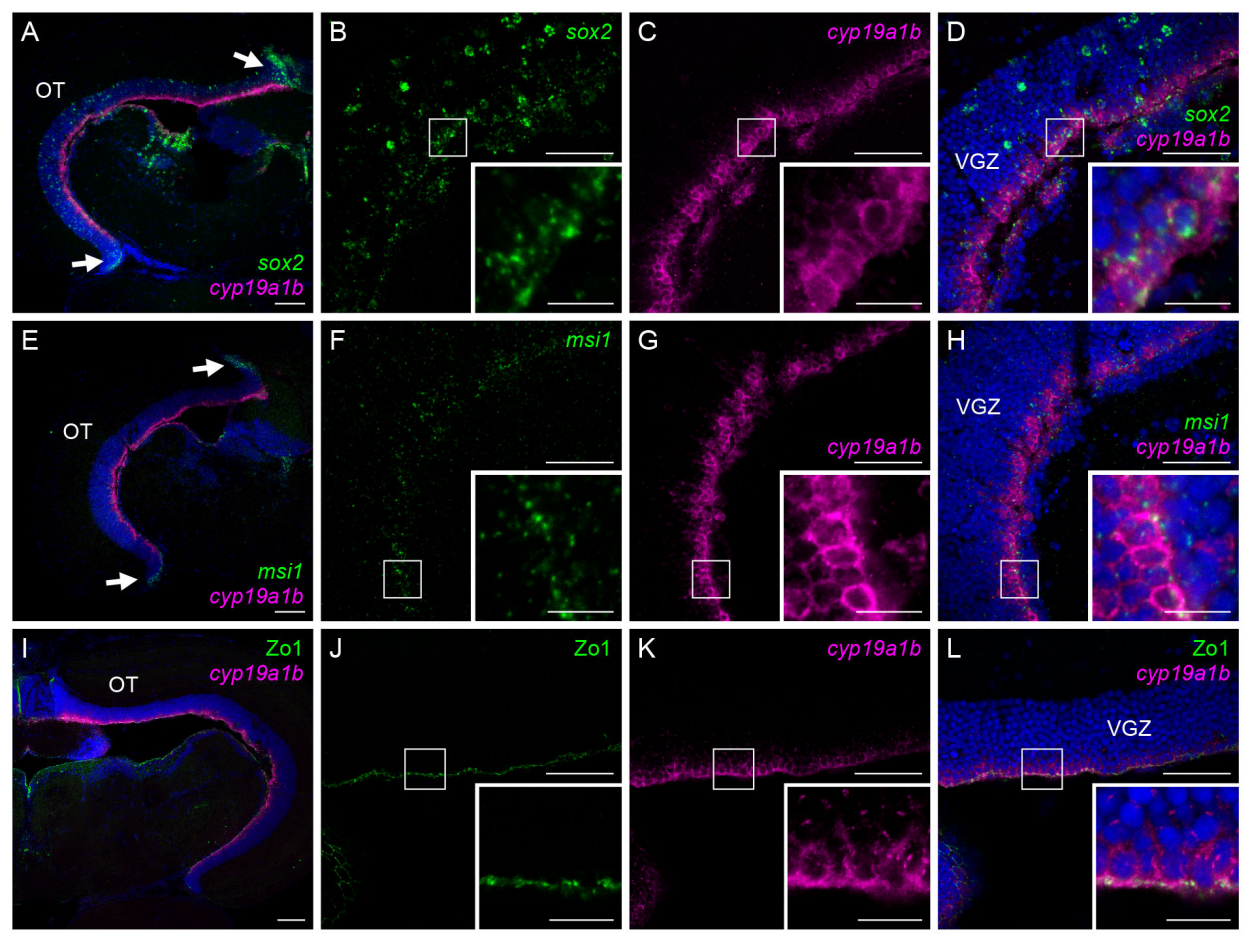
The tectal aromatase-expressing cells express genes indicative of multipotency and exhibit cell polarity. Coexpression of *cyp19a1b* and the genes indicative of multipotency, *sox2* (A–D) and *msi1* (E–H), and epithelial/radial glial cell polarity marker, Zo1 (I–L), in female medaka optic tectum. (A) Expression of *sox2* and *cyp19a1b* in the optic tectum overall. Arrows indicate the peripheral growth zone. Nuclei were stained with DAPI. (B) *sox2* expression in the VGZ. (C) *cyp19a1b* expression in the same field as panel B. (D) Merged image of panels B and C along with DAPI staining. (E) Expression of *msi1* and *cyp19a1b* in the whole optic tectum. Arrows indicate the peripheral growth zone. Nuclei were stained with DAPI. (F) *msi1* expression in the VGZ. (G) *cyp19a1b* expression in the same field as panel F. (H) Merged image of panels F and G along with DAPI staining. (I) Expression of Zo1 and *cyp19a1b* in the whole optic tectum. Nuclei were stained with DAPI. (J) Zo1 expression in the VGZ. (K) *cyp19a1b* expression in the same field as panel J. (L) Merged image of panels J and K along with DAPI staining. Small boxes in panels B–D, F–H, and J–L indicate regions shown at higher magnification in the insets in the lower right-hand corner of the respective panels. Scale bars, 100 µm (A, E, I), 50 µm (B–D, F–H, J–L), and 10 µm (all insets). OT, optic tectum; VGZ, ventricular gray zone of the optic tectum.

Two genes indicative of multipotency, *sox2* and *msi1*, displayed similar expression patterns in the medaka optic tectum (Figure 3A–H). The most prominent expression of both genes was observed in the peripheral growth zone (Figure 3A, E, arrows). Their expression was also found to be spread along the VGZ, with the signals for *sox2* being slightly broader and more intense than those for *msi1* (Figure 3B, F). Expression of *sox2* and *msi1* was scattered throughout all layers of the VGZ, whereas that of *cyp19a1b* was restricted to a thin layer in the ventricular surface (Figure 3B–D, F–H). Despite this difference in distribution, most of the *cyp19a1b*-expressing cells were found to express both *sox2* and *msi1* (Figure 3B–D, F–H, insets).

Immunostaining for Zo1, an epithelial/radial glial cell polarity marker, labeled the ventricular surface of the VGZ (Figure 3I–L). The signals for Zo1 were evenly spread over the surface (Figure 3J) and covered all *cyp19a1b*-expressing cells facing the ventricle (Figure 3J–L). Examination at higher magnification showed that the Zo1 signals were exclusively located on the apical surface of the *cyp19a1b*-expressing cells, thus demonstrating their cell polarity (Figure 3J–L, insets).

### Female-specific aromatase-expressing cells in the medaka optic tectum were immature radial glial cells

**Figure 4:**
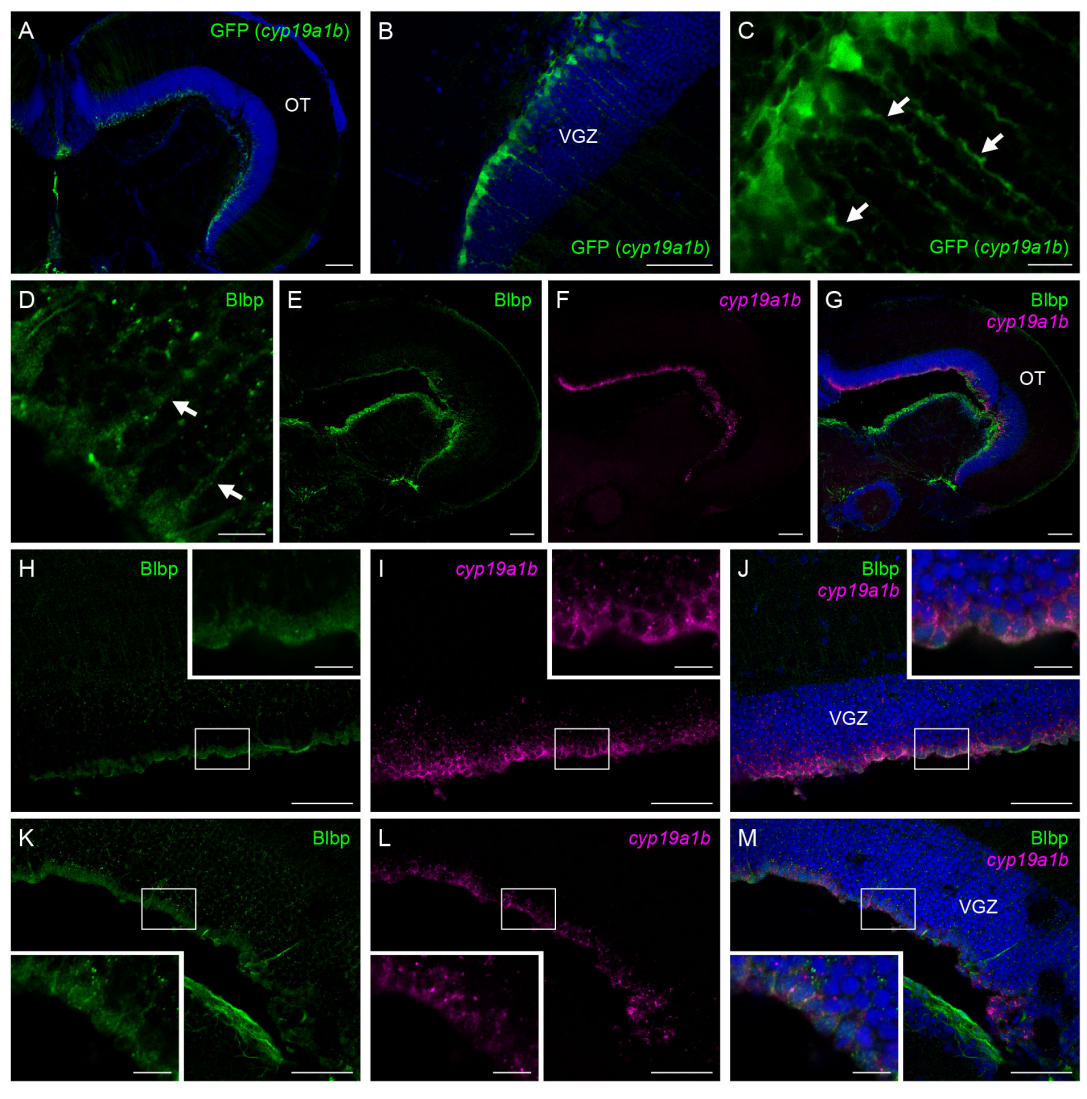
The tectal aromatase-expressing cells are immature radial glial cells. Morphology of female-specific *cyp19a1b*-expressing cells in the medaka optic tectum as revealed by imaging of *cyp19a1b*-GFP transgenic medaka (A–C) and expression of Blbp in these cells (D–M). (A) GFP fluorescence in the whole optic tectum in *cyp19a1b*-GFP transgenic medaka. Nuclei were stained with DAPI. (B) GFP fluorescence in the VGZ shown at higher magnification than panel A. (C) Highly polarized morphology of GFP-positive cells with long cytoplasmic processes (indicated by arrows). (D) Blbp-positive cells exhibiting a radial morphology with long cytoplasmic processes (indicated by arrows). (E) Blbp expression in the whole optic tectum. (F) *cyp19a1b* expression in the same field as panel E. (G) Merged image of panels E and F along with DAPI staining. (H) Blbp expression in the peripheral portion of the optic tectum. (I) *cyp19a1b* expression in the same field as panel H. (J) Merged image of panels H and I along with DAPI staining. (K) Blbp expression in the central portion of the optic tectum. (L) *cyp19a1b* expression in the same field as panel K. (M) Merged image of panels K and L along with DAPI staining. Small boxes in panels H–M indicate regions shown at higher magnification in the insets in respective panels. Scale bars, 100 µm (A, E–G), 50 µm (B, H–M), and 10 µm (C, D, all insets). OT, optic tectum; VGZ, ventricular gray zone of the optic tectum.

GFP fluorescence imaging in the optic tectum of the *cyp19a1b*-GFP transgenic medaka allowed delineation of the shape of female-specific *cyp19a1b*-expressing cells. Strong GFP fluorescence was detected in the VGZ of the female tectum, as was endogenous *cyp19a1b* expression (Figure 4A, B). The GFP-labeled cells were highly polarized, extending long cytoplasmic processes (Figure 4C).

In addition to Gfap, the presence of another radial glial marker Blbp, whose expression is initiated immediately after the differentiation of radial glial cells in rodents [26,27], was examined in the *cyp19a1b*-expressing cells. As expected, Blbp-positive cells exhibited a radial glial morphology with cell bodies in the ventricular surface and long processes that extended to the pial surface (Figure 4D). They spread out more broadly and uniformly throughout the VGZ than Gfap-positive cells and overlapped largely with the *cyp19a1b*-expressing cells, although negligible or weak Blbp staining was observed in the peripheral-most region of the VGZ (Figure 4E–G). In contrast to Gfap, Blbp was coexpressed with *cyp19a1b* over the VGZ, whether in the peripheral (Figure 4H–J) or central (Figure 4K–M) portion of the optic tectum.

The above series of histological analyses were also carried out with the male tectum. The expression pattern of each transcript/protein in the male tectum was comparable to that in the female tectum, except that *cyp19a1b* expression was not detected in the male tectum (data not shown).

### Sex differences in the expression of cell life cycle-related genes in the medaka optic tectum and sex-specific size differences of the medaka optic tectum

**Figure 5:**
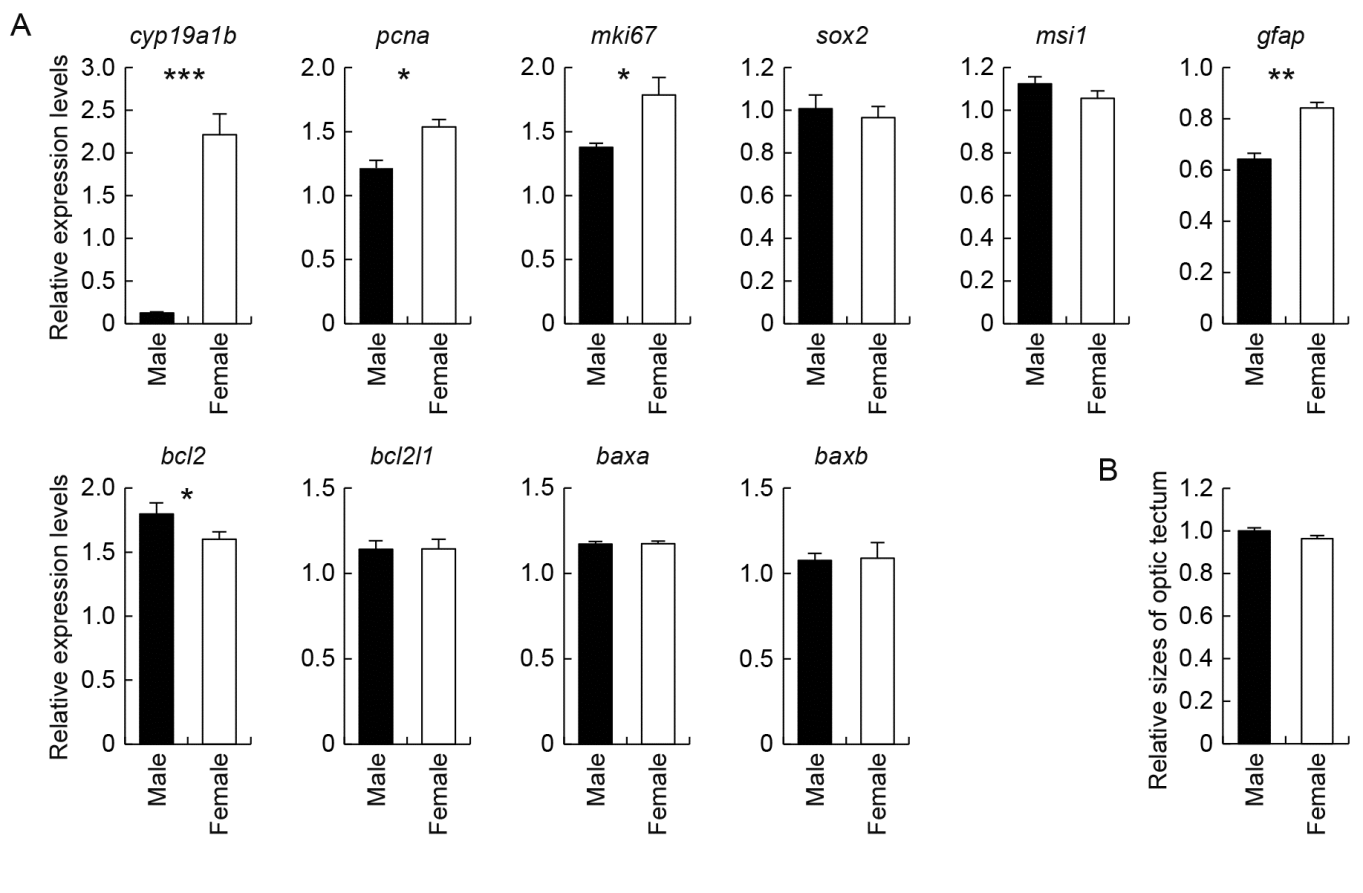
Sex differences in the tectal expression of cell life cycle-related genes and the tectal size. Examination of sex differences in the expression of cell life cycle-related genes as well as *cyp19a1b* in the medaka optic tectum (A) and its size (B). (A) Examination of sex differences in the expression of *cyp19a1b*, proliferation markers, *pcna* and *mki67*, genes indicative of multipotency, *sox2* and *msi1*, mature radial glial marker, *gfap*, anti-apoptotic genes, *bcl2* and *bcl2l1*, and pro-apoptotic genes, *baxa* and *baxb*, in the medaka optic tectum. Values are given relative to the level of expression in the whole brain of males, which was arbitrarily set to 1. *, *p* < 0.05; **, *p* < 0.01; ***, *p* < 0.001. (B) Examination of sex differences in the optic tectum size in medaka. No significant difference was detected between males and females (*p* = 0.12).

Possible sex differences in the cell life cycle in the medaka optic tectum were assessed by comparing the expression levels of cell life cycle-related genes in the male and female tectum by real-time PCR analysis (Figure 5A). We first confirmed that *cyp19a1b* was abundantly expressed in the female tectum, whereas its expression was very low, if not entirely absent, in the male tectum. The expression levels of *pcna* and *mki67*, both of which served as cell proliferation markers, were significantly higher in the female tectum than the male tectum: both genes showed about 1.3-fold higher expression in females. Similarly, the expression of *gfap*, indicative of radial glial maturation, was about 1.3-fold higher in the female tectum. As opposed to these female-biased genes, *bcl2*, an anti-apoptosis gene, exhibited slightly but significantly (1.1-fold) higher expression in the male tectum than the female tectum. No significant sex differences were observed for the expression of *sox2*, *msi1*, *bcl2l1*, *baxa*, or *baxb*.

Next, the possibility that the size of the medaka optic tectum was sexually dimorphic was tested by measuring the size ratio of the optic tectum to the telencephalon and comparing it between males and females. The result showed that there was no significant sex difference in the relative optic tectum size (Figure 5B).

### Effects of estrogens on the expression of sexually dimorphic genes related to the cell life cycle in the medaka optic tectum

**Figure 6:**
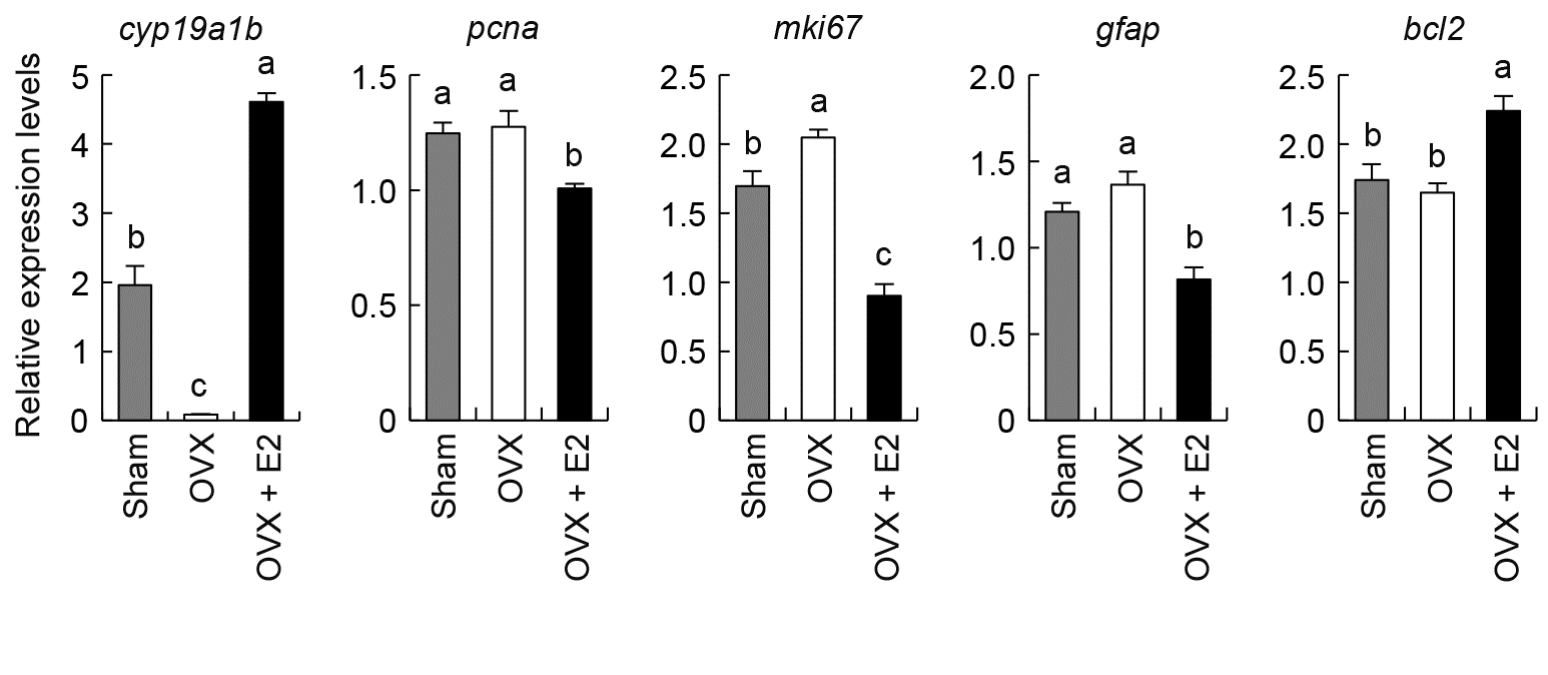
Effects of estrogens on the tectal expression of cell life cycle-related genes. Effects of ovariectomy (OVX) and E2 treatment on the expression of *cyp19a1b* and the cell life cycle-related genes differentially expressed between the sexes (*pcna*, *mki67*, *gfap*, and *bcl2*) in the optic tectum. Values are given relative to the level of expression in the whole brain of males, which was arbitrarily set to 1. Groups with different alphabetical characters are significantly different (*p* < 0.05).

We subsequently examined the possibility that estrogens, the product of aromatase activity, contributed to the sexually dimorphic expression of cell life cycle-related genes in the medaka optic tectum (Figure 6). We began by examining the expression of *cyp19a1b* and found that its expression in the optic tectum was substantially reduced by ovariectomy and was restored by treatment with E2. Ovariectomy elevated *mki67* expression, while causing no significant changes in the expression of *pcna*, *gfap*, or *bcl2*. E2 treatment led to a significant reduction in the expression of *pcna*, *mki67*, and *gfap* and, in contrast, a significant increase in the expression of *bcl2*.

## Discussion

We have recently demonstrated the presence of female-specific aromatase-expressing cells in the medaka optic tectum [16]. It is generally accepted that in the teleost brain, aromatase is expressed in mature radial glial cells with proliferative activity [6–9,12–14]. This concept has been repeatedly verified by analyzing the forebrain, including the preoptic area and hypothalamus, but no studies have addressed the property of aromatase-expressing cells in the optic tectum. Moreover, recent studies have indicated that radial glial cells and proliferating cells are discernible as separate cell populations in the teleost optic tectum [17,18]. Therefore, we investigated which cell population was the source of female-specific aromatase expression in the medaka optic tectum in the present study. Unexpectedly, we found that the majority of female-specific cells expressing the aromatase gene, *cyp19a1b*, expressed neither Gfap nor Pcna/*mki67*, indicating that they are neither mature radial glial cells nor proliferating cells. This finding is in contrast to what has been generally observed for the teleost brain, where aromatase is consistently coexpressed with these markers.

The question then arose as to what cell population expressed aromatase female-specifically in the medaka optic tectum. We found that *cyp19a1b* was coexpressed with *sox2* and *msi1*, suggesting that the aromatase-expressing cells are multipotent stem-like cells. Coexpression of *cyp19a1b* and Zo1 was also found, suggesting that they are polarized neuroepithelial/radial glial cells as well. These findings indicate that female-specific aromatase-expressing cells most likely constitute a subpopulation of multipotent neuroepithelial- or radial glial-stem cells. We further showed that these cells, when tagged transgenically with GFP, displayed a highly polarized morphology with long cytoplasmic processes and that most of them expressed Blbp, an earlier marker of radial glial differentiation than Gfap [26–29]. Taken together, these data provide evidence that female-specific aromatase-expressing cells in the medaka optic tectum represent a unique subset of post-proliferative immature radial glial cells that presumably function as neural stem cells.

This scenario is in reasonable agreement with the relative distributions of *cyp19a1b* expression and those of various cell-type markers as revealed by this study. The teleost optic tectum is dome-shaped and continues to grow by the addition of sequential rings of new cells at the marginal portion called the peripheral growth zone [30–32]. Consistent with these data, here we identified Pcna/*mki67*-positive cells in the peripheral growth zone. We also found that *cyp19a1b* expression appeared most intense in the vicinity of this zone and gradually diminished toward the central portion of the optic tectum, where Gfap-positive cells were densely distributed. These lines of evidence indicate that the aromatase-expressing cells are in a transient state after cellular proliferation and yet before radial glial maturation, along a peripheral-to-central sequence in the optic tectum cell development.

It should be noted that we observed *sox2* and *msi1* expression in a small population of the *cyp19a1b*-expressing cells. In the zebrafish optic tectum, *gfap*-expressing mature radial glial cells have been shown to express these stem cell markers and be capable of proliferating, though at a much slower rate than cells in the peripheral growth zone [17,33]. It may thus be possible that the aromatase-expressing immature radial glial cells retain stem cell-like properties and the ability to reenter the cell cycle. In line with this possibility, there is evidence that mature astrocytes in the mammalian brain can dedifferentiate under certain conditions to become radial glial cells with proliferative potential, indicating that even mature astrocytes have the ability to reenter the cell cycle [34].

The finding that a unique subset of glial cells expresses aromatase in a female-specific manner during their early phase of differentiation in the medaka optic tectum led us to hypothesize that this aromatase expression causes some sex differences in the optic tectum cell fate determination. This conclusion seems possible when considering that, in the sexual differentiation process of the rodent brain, estrogens locally produced by aromatase bring about sexual dimorphisms in the proliferation, differentiation, and death of brain cells [2]. In the brain of teleosts, however, the role of local estrogen synthesis in sexual differentiation remains unknown, and, to our knowledge, virtually no studies have assessed sex differences in the cell life cycle. In the present study, we examined possible sex differences in the expression of cell life cycle-related genes in the medaka optic tectum and, as expected, some of the genes examined did exhibit sexually dimorphic expression levels: the female tectum exhibited higher expression of the genes indicative of cell proliferation, *pcna* and *mki67*, and radial glial maturation, *gfap*, and lower expression of an anti-apoptotic gene, *bcl2*, than did the male tectum. These data suggest a female-biased acceleration of cell proliferation, differentiation, and death in the medaka optic tectum. The subsequent finding that the optic tectum size was not sexually dimorphic further suggests an equivalent ratio of cell proliferation to cell death between the sexes as a consequence of the acceleration of both these events in females. It seems plausible that these newly found possible sex differences in the cell life cycle may lead to some sex differences in optic tectum function. Given that the optic tectum is implicated in sensorimotor coordination [35], these may underlie sex differences in receiving some sensory inputs and/or sending out motor commands for orientation to stimuli. Examining this possibility would be an interesting and important direction for future research.

It is reasonable to assume that the sexually dimorphic expression of the cell life cycle-related genes observed stems from the effects of estrogens. Indeed, it has been shown that E2 stimulates mitotic activity of cells in diverse brain regions of rodents [36–40]. In the arcuate nucleus of the rat brain, astrocytes exhibit a more differentiated morphology in males than females, due to the perinatal action of E2 abundantly synthesized in the male brain [41]. In addition, sex differences in apoptosis mediated by estrogenic modulation of survival and death factors including *Bcl2* are present in the perinatal rodent brain, and this contributes to sex differences in the size or cell density of particular brain nuclei [42–45].

Our results indeed demonstrated the ability of E2 to modulate the expression of the cell life cycle-related genes that were differentially expressed between the sexes in the optic tectum. However, the effect of E2 we observed was in the opposite direction to what we predicted: E2 suppressed the expression of *pcna*, *mki67*, and *gfap* and stimulated the expression of *bcl2*, suggesting a role for estrogens in the restraint of the life cycle of tectal cells. Hence, it seems that E2 serves not to increase but rather to diminish sex differences in the expression of these genes, while simultaneously stimulating aromatase expression. A similar result has recently been obtained in the telencephalon and hypothalamus of zebrafish, where E2 exerted a suppressive effect on cell proliferation [46]. Although this discrepancy between sex differences and the effect of estrogens cannot be explained at present, it seems possible that female-specific aromatase expression and resultant estrogen production in the optic tectum play a role to compensate for sex differences in the life cycle of tectal cells, which are caused by an as yet unidentified mechanism. Alternatively, the ability of estrogens to either promote or inhibit the life cycle of tectal cells may depend on its concentration and/or the physiological status of the cells. One may suspect that the conspicuous sex difference in *cyp19a1b* mRNA expression in the optic tectum does not necessarily result in a parallel sex difference in aromatase enzyme activity in this region, especially considering that aromatase might undergo post-transcriptional and post-translational modifications. However, this seems unlikely as there is evidence that aromatase activity in medaka brain sections containing the optic tectum is confined almost exclusively to females [47]. Further studies are needed to determine the exact role of female-specific tectal aromatase expression and consequent local synthesis of estrogens in the control of the cell life cycle.

In summary, the present study demonstrated that a transient population of post-proliferative immature radial glial cells in the medaka optic tectum express aromatase in a female-specific manner and that there are sex differences in the expression of genes linked to cell proliferation, glial maturation, and apoptosis in the medaka optic tectum, suggesting a female-biased acceleration of the cell life cycle. Our data also suggest that female-specific aromatase expression and resultant estrogen production have an impact on the life cycle of tectal cells, whether stimulatory or inhibitory. The medaka optic tectum should provide a unique and useful model system to study the role of aromatase/estrogens in brain sexual differentiation because it shows clear sex differences: female-specific occurrence of aromatase-expressing cells and sexually dimorphic expression of a series of genes involved in the cell life cycle. Future studies using this system will further our understanding of sex differences in brain function and neurogenesis as well as the importance of aromatase/estrogens in these issues.
